# Gene‐Environment Interactions in Late‐life Cognitive Functioning: The Impact of Polygenic Scores and Early Life Trauma in African American Older Adults

**DOI:** 10.1002/alz70855_106877

**Published:** 2025-12-25

**Authors:** Soohyun Park, Anthony H Mark, Eilis W Reardon, Regan E. Patrick, Brent P. Forester

**Affiliations:** ^1^ Tufts University School of Medicine, Boston, MA, USA; ^2^ Tufts Medical Center, Boston, MA, USA; ^3^ Harvard Medical School, Boston, MA, USA; ^4^ McLean Hospital, Belmont, MA, USA

## Abstract

**Background:**

African Americans (AAs) experience disproportionately higher rates of Alzheimer's Disease and Related Dementias and significant disparities in access to diagnosis and care for cognitive health compared to White individuals. Although studies have investigated the effects of genetic risk (e.g., APOE) and early‐life trauma on late‐life cognitive functioning in AAs, the role of polygenic scores for general cognition (PGS‐gc)—*the aggregated effects of numerous genetic loci to estimate genetic predisposition for cognitive functioning*— and their interplay with early‐life trauma on cognitive functioning in older AAs remains understudied. This study investigated 1) the main and additive effects of PGS‐gc and early‐life trauma on cognitive functioning and 2) whether early‐life trauma moderates the effect of PGS‐gc on cognitive functioning in older AAs, leveraging a nationally representative, longitudinal dataset.

**Method:**

Growth curve modeling was performed on 1,174 African ancestry adults aged 50+ from the 1996‐2020 waves (13 waves) of the Health and Retirement Study. Cognitive functioning was measured by a total cognition score. Genetic predisposition for cognitive functioning was measured via PGS‐gc, and early‐life trauma was measured using 10 items assessing adverse childhood experiences.

**Result:**

Covariate‐adjusted analyses found a positive and significant association between PGS‐gc and cognitive functioning (z = 2.59, *p* < .05, 95% CI [0.06, 0.47]), indicating that higher PGS‐gc was associated with better cognitive performance. Family loss before age 16 was significantly associated with lower cognitive functioning, even after accounting for PGS‐gc (z = ‐2.49, *p* < .05, 95% CI [‐0.95, ‐0.11]). Moreover, parental physical/substance abuse before age 18 significantly moderated the relationship between PGS‐gc and cognitive functioning (z = ‐2.53, *p* < .05, 95% CI [‐1.14, ‐0.14]), suggesting that the positive association between PGS‐gc and cognitive functioning was attenuated in those who experienced such trauma.

**Conclusion:**

These longitudinal findings are among the first to elucidate a gene‐by‐environment interaction on cognitive functioning in older AAs, suggesting that early‐life trauma (e.g., parental physical or substance abuse) significantly moderates and weakens the positive association between polygenic predisposition and late‐life cognitive functioning. These results underscore the critical need for trauma prevention and trauma‐informed care for racially/ethnically minoritized older AAs to promote late‐life cognitive health and address cognitive disparities.

